# Hemorphins Targeting G Protein-Coupled Receptors

**DOI:** 10.3390/ph14030225

**Published:** 2021-03-07

**Authors:** Mohammed Akli Ayoub, Ranjit Vijayan

**Affiliations:** 1 Department of Biology, College of Science, United Arab Emirates University, P.O. Box 15551 Al Ain, United Arab Emirates; 2 Zayed Center for Health Sciences, United Arab Emirates University, P.O. Box 15551 Al Ain, United Arab Emirates

**Keywords:** hemorphins, GPCR, hemoglobin, opioid receptors, RAS, AngII, AT1R, ACE

## Abstract

Hemorphins are short peptides produced by the proteolysis of the beta subunit of hemoglobin. These peptides have diverse physiological effects especially in the nervous and the renin-angiotensin systems. Such effects occur through the modulation of a diverse range of proteins including enzymes and receptors. In this review, we focus on pharmacological and functional targeting of G protein-coupled receptors (GPCRs) by hemorphins and their implication in physiology and pathophysiology. Among GPCRs, the opioid receptors constitute the first set of targets of hemorphins with implication in analgesia. Subsequently, several other GPCRs have been reported to be directly or indirectly involved in hemorphins’ action. This includes the receptors for angiotensin II, oxytocin, bombesin, and bradykinin, as well as the human MAS-related G protein-coupled receptor X1. Interestingly, both orthosteric activation and allosteric modulation of GPCRs by hemorphins have been reported. This review links hemorphins with GPCR pharmacology and signaling, supporting the implication of GPCRs in hemorphins’ effects. Thus, this aids a better understanding of the molecular basis of the action of hemorphins and further demonstrates that hemorphin-GPCR axis constitutes a valid target for therapeutic intervention in different systems.

## 1. Introduction

**Figure 1:**
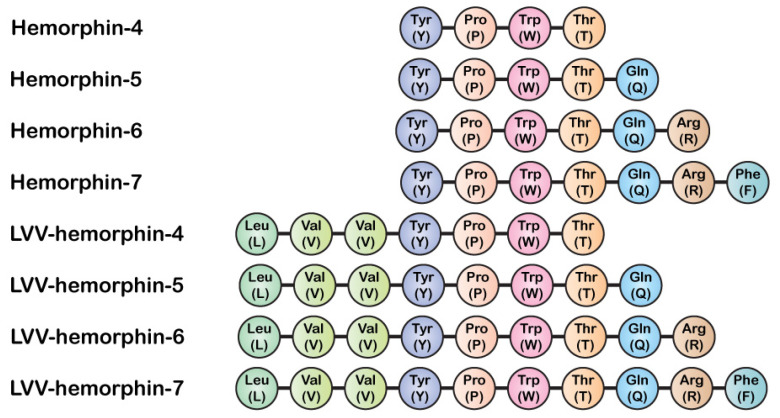
Hemorphin peptides and their amino acid sequence.

Hemoglobin (Hb)-derived peptides constitute interesting bioactive molecules with a role in both physiology and pathophysiology [1,2,3,4]. Among these Hb-derived peptides, hemorphins have been extensively studied in various contexts including the nervous, vascular, and endocrine systems [1,2,3,4]. Indeed, hemorphins have been shown to have various biological activities, including effects on spatial learning [5], analgesia [6,7,8,9,10,11,12], antihypertension [3,13,14,15,16,17,18], constriction of coronary vessels and platelet aggregation [19], and inflammation [10,11]. Hemorphins are endogenous peptides released during the sequential cleavage of hemoglobin protein and initially reported as atypical opioid peptides [2,3,4,20,21,22,23,24,25]. They have also been produced in vitro by the proteolytic cleavage of the beta chain of hemoglobin protein [26]. Hemorphin peptides vary from 4 to 10 amino acids (Figure 1). Hemorphin-4 was the first hemorphin to be identified and characterized from bovine blood [4]. Their biogenesis is very complex and involves many enzymes and proteolytic pathways and their tissue-specific production and concentration are still not fully understood [4]. In terms of sequence, hemorphin peptides share a core tyrosine-proline-tryptophan-threonine (YPWT) tetrapeptide. N- and C-terminal extensions of this core sequence have been isolated from human and bovine tissues [2,23,25] (Figure 1). N-terminal extensions are predominantly hydrophobic in nature characterized by the presence of leucine and valine residues. Longer isoforms such as LVV-hemorphin-7 and VV-hemorphin-7 have been shown to be the most stable in vitro [24] and abundant in the bovine brain [25,27].

Hemorphins present various physiological functions in different systems and tissues. The versatile hemorphins have been documented to target a number of different enzymes and receptors in distinct pathways [3,4]. Several of these belong to the renin-angiotensin system (RAS) and the kinin-kallikrein system (KKS), with key relevance to blood pressure regulation and therefore hypertension [28], and has led to the seemingly opposing action of hemorphins on the enzyme angiotensin-converting enzyme (ACE) [14] and angiotensin II (AngII) [29] receptor (AT1R) as well as the bradykinin receptor. The ACE inhibitory activity of hemorphins has been linked to the N-terminal hydrophobic extensions to the core tetrapeptide [30,31,32]. Along with angiotensin, LVV-hemorphin-7 has also been shown to bind to and competitively inhibit the multifaceted serine exopeptidase dipeptidyl peptidase-4 (DPP4) [33], which is responsible for cleaving a number of bioactive peptides and is therefore relevant in a number of independent pathways including RAS and hyperglycemia [34]. Hemorphins have been demonstrated to modulate the activity of calcineurin and Ca^2+/^calmodulin dependent systems with potential implication in the immune system and the brain [35]. Hemorphins have also been demonstrated to suppress the proliferation of tumor cells and significantly decrease in hemorphin peptides were observed in the serum of breast cancer patients. Hence, while the precise mechanism is unknown, hemorphins also have the potential to be both a biomarker and a therapeutic agent for some forms of cancer [36]. Clearly, hemorphins modulate the activity of structurally and functionally diverse enzymes and receptors by targeting both orthosteric and allosteric sites of these proteins.

**Figure 2:**
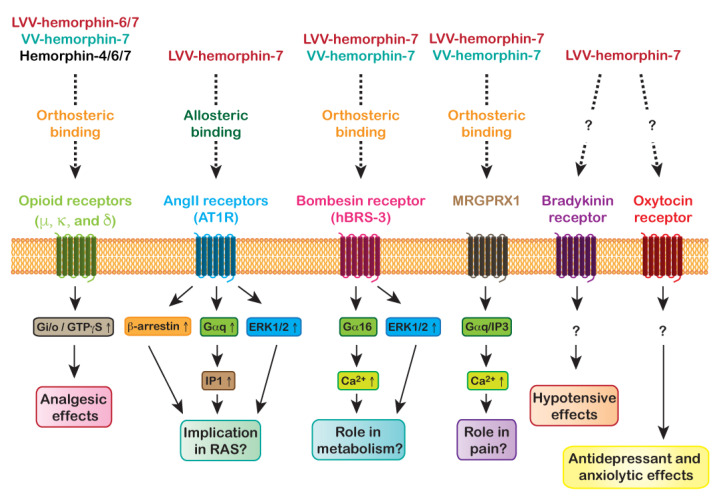
GPCRs targeted by the hemorphins along with their mode of binding, signaling and plausible implication in physiology and pathophysiology.

In this review, we focused on GPCRs as one of the important molecular targets of hemorphins. It provides an overview of established targets like the opioid receptors and other GPCR members to our recent novel findings related to hemorphins targeting AT1R (Figure 2) (Table 1). Overall, data on hemorphins targeting GPCRs support the existence of both orthosteric as well as allosteric modulation of GPCRs. This indicates the diversity of hemorphins’ mode of action on GPCRs illustrating the complexity of hemorphin-GPCR axis and its implication in physiology and pathophysiology.

## 2. Hemorphins and Opioid Receptors

Opioid receptors constitute the first GPCR family identified as a pharmacological target of hemorphins, using in vitro and in vivo assays, with both opiate and anti-opiate activities [6,7,8,9,22,23,26,37,38,39,40,41]. The targeting of the opioid receptors was physiologically linked to the analgesic effects of hemorphins [6,7,8,9,10,11,12]. Indeed, radioligand binding experiments showed a partial to full binding of various forms of hemorphin peptides (hemorphin-4, LVV-hemorphin-6, hemorphin-6, LVV-hemorphin-7, VV-hemorphin-7) in a competitive manner with the endogenous opioid-related peptides such as enkephalins and dynorphins [6,26,39,40,41]. This includes μ-, κ-, and δ-opioid receptors with differential affinities between the different forms of hemorphin peptides towards these receptor subtypes. This was true for both the synthetic as well as the endogenous hemorphin peptides purified from bovine blood and brain [6,26,39,40,41]. Opioid peptide-based studies have alluded to the relevance of the N-terminal tyrosine, which is also characteristic of the core hemorphin tetrapeptide. N-terminal extensions of the core sequences in hemorphins have been shown to affect the potency and affinity of hemorphins [26]. In silico results suggested hemorphins interact with residues of Asp149, Trp320 and Tyr328 of μ-opioid receptors [3]. While Asp149 and Tyr328 are conserved across the opioid receptors Trp320 is not. This could account for the differential affinities towards the subtypes since Trp320 and Tyr328 have both been demonstrated to influence opiate ligand bias [42].

From a pharmacological point of view, hemorphins showed agonistic action at the opioid receptors. For instance, the nanopeptide, LVV-hemorphin-6, its shorter form hemorphin-6 that lacks the N-terminal hydrophobic residues, and LVV-hemorphon-7 were shown to significantly activate opioid receptors in electrically induced contractions in rat vas deferens (RVD) and guinea-pig ileum (GPI) assays [38,40]. This activity of hemorphin peptides was reversed by the opiate antagonist naloxone [40]. Moreover, these hemorphins decreased the inhibitory action of β-endorphin with IC_50_ values of 37 and 73 μM for hemorphin-6 and LVV-hemorphin-6, respectively. Hemorphin-6 at 100 μM also antagonized the inhibitory action of normorphine, but not that of dynorphin A [38]. Similarly, VV-hemorphin 7 has been shown to compete reversibly with the binding of subtype selective opioid radioligands, [^3^H]-DAMGO (μ-selective peptide), heterocyclic [^3^H]-cyprodime (κ-selective heterocyclic analogue), and [^3^H]-Ile5,6-deltorphin and [^3^H]-TIPP (δ-selective peptides) analyzed in rat brain membrane preparations [39]. Together, these observations indicate the orthosteric binding and activation of the opioid receptors by hemorphin peptides. Despite the evidence for hemorphin peptides binding to different opioid receptors, very little has been reported regarding the activation of these receptors, their cognate heterotrimeric G proteins and downstream signaling pathways. VV-hemorphin 7 has been demonstrated to promote partial [^35^S]-GTPγS incorporation (~75%) in a dose-dependent manner in a rat brain homogenate indicative of opioid receptor activating its cognate G proteins, probably Gi/o proteins [39]. However, such opioid receptor-G protein coupling promoted by hemorphin peptides was not demonstrated at the level of downstream signaling pathways. However, a specific G protein-dependent pathway and its link with the analgesic effect of hemorphins has not been fully investigated. This constitutes an interesting aspect for any further studies on the pharmacological targeting of opioid receptors by hemorphins. The diversity of hemorphins along with the multiple signaling pathways of the opioid receptors (G proteins, β-arrestins, etc.) might provide the rationale for investigating in detail the pharmacology of hemorphin-opioid receptor pairs in the “hot” context of biased signaling/functional selectivity of GPCR pharmacology [43].

## 3. Hemorphins and Angiotensin II Receptor

The role of hemorphin peptides in RAS has been documented in many in vitro and in vivo studies indicating antihypertensive activity and implication in the vascular system [3,13,14,15,16,17,18]. At the molecular level, this has been attributed to the specific binding and inhibition of the key enzyme, ACE, as shown in vitro and in silico [13,30,31,32].

Recently, we reported for the first time the targeting of AngII type 1 receptor (AT1R) by LVV-homorphin-7 in vitro using the human embryonic kidney (HEK293) cells [29]. We revealed a positive allosteric modulation (PAM) of AT1R by LVV-hemorphin-7. Interestingly this was the first study to report the allosteric modulation of a GPCR by a hemorphin peptide [29]. The PAM action of LVV-hemorphin-7 was revealed using bioluminescence resonance energy transfer (BRET) technology in live HEK293 cells and characterized by a potentiation in AngII/AT1R-promted Gαq protein coupling, β-arrestin recruitment, and the activation of the extracellular signal-regulated kinases (ERK1/2) [29]. Furthermore, we demonstrated the specific binding of LVV-hemorphin-7 on AT1R in vitro using the Nano-BRET binding assay in live HEK293 cells as well as in silico by molecular docking and molecular dynamics simulations suggesting the existence of an allosteric binding site for LVV-hemorphin-7 in the intracellular loops of AT1R [44]. Our docking and simulations data suggested that LVV-hemorphin-7 interacted with specific residues in the intracellular loops 2 and 3 and the cytoplasmic end of transmembrane helices 3 and 6 (TM3 and TM6) of AT1R [44]. This is consistent with the specific saturation-binding assay demonstrating the non-competitive binding of LVV-hemorphin-7 on AT1R. Indeed, LVV-hemorphin-7 significantly potentiated the affinity of AngII by decreasing its K_d_ by 2.6 fold without affecting its maximal binding (B_max_) indicative of a PAM profile of the peptide on AT1R [44]. The positive effect of LVV-hemorphin-7 on AngII’s affinity was supported by our previous functional data on AT1R signaling [29]. Molecular dynamics simulations indicated conformational changes in the structure that permitted deeper embedding of the C-terminal of AngII in the orthosteric site of AT1R in the presence of LVV-hemorphin-7 when compared to the structure without LVV-hemorphin-7, alluding to an allosteric effect [44]. Such an allosteric mechanism was recently demonstrated using AT1R-stabilizing nanobodies that target the intracellular loop 2 of AT1R, thereby enhancing the binding affinity of AngII and its potency [45]. Furthermore, the allosteric binding of LVV-hemorphin-7 in the intracellular regions of AT1R is consistent with the allosteric modulation of GPCRs through the targeting of their intracellular regions by small molecule ligands [46,47,48] and cell-penetrating lipidated peptides termed pepducins [49,50]. Interestingly, a partial agonistic effect of LVV-hemorphin-7 was observed on AT1R mediated by the activation of the extracellular signal–regulated kinases (ERK1/2) but not on AT1R/Gαq-mediated inositol phosphate production suggesting a biased effect [29]. While extracellular and orthosteric binding sites have been explored in some detail, potentially pharmacologically-relevant intracellular sites have received limited attention [51,52]. Positive and negative allosteric modulators have been reported targeting these sites. Given the complexity and diversity of GPCRs, this further opens up the possibilities for the identification and design of allosteric modulators targeting intracellular loops.

If the PAM action of LVV-hemorphin-7 on AT1R revealed in HEK293 cells also occurs in vivo, this suggests hypertensive effects of the hemorphin peptide. While this may not be consistent with the well-known antihypertensive properties of hemorphins by its opposing action on RAS via ACE inhibition and on the KKS by potentiating bradykinin action [3,13,14,15,16,17,18], it indicates the complexity of RAS and the implication of hemorphins and AT1R in its regulation. This aspect was largely discussed with a few speculations in our recent work on AT1R [29].

## 4. Hemorphins and the Bradykinin Receptor

As stated earlier, hemorphins play a major role in the regulation of blood pressure by targeting different components of RAS and KKS [3,13,14,15,16,17,18]. One of the notable effects is the potentiation of bradykinin action in vivo mediated by these bioactive peptides, isolated from dog pancreas and sheep brain, on the blood pressure of anaesthetized rats [15]. Among these peptides, LVV-hemorphin-7 was very active similar to the synthetic form of the peptide [15]. Indeed, 60 μg of LVV-hemorphin-7 increased by 31% the hypotensive effect of bradykinin at 0.5 μg [15]. In addition, this observation is consistent with the antihypertensive properties of hemorphins through the inhibition of ACE [13,30,31,32]. However, the study did not address the possible pharmacological and functional action of hemorphins directly on bradykinin receptors. This is yet to be investigated either in vivo or in vitro. Moreover, it is not clear which bradykinin receptor subtype, B1R or B2R, was targeted by the hemorphin peptides. Nonetheless, the positive action of LVV-hemorphin-7 on bradykinin-mediated response suggests an allosteric interaction between these two peptides. This is interestingly similar to the PAM action of LVV-hemorphin-7 observed on AT1R in HEK293 cells [29]. Therefore, this suggests a similar mode of action (PAM) of hemorphins on key receptors of RAS and KKS, AT1R and bradykinin receptor, respectively [29]. This further supports the complex role that hemorphins play in the regulation of blood pressure by targeting diverse molecular targets. Further in vitro and in vivo investigations considering both RAS and KKS are necessary to dissect the different pathways involved in the targeting of bradykinin receptor by hemorphins.

## 5. Hemorphins and the Bombesin Receptor

The human bombesin receptor subtype 3 (hBRS-3) is another GPCR shown to be pharmacologically and functionally sensitive to hemorphin binding as demonstrated in both endogenously and transiently expressing cells [53]. HBRS-3, originally classified as an orphan receptor, shares high amino-acid sequence homology with the human neuromedin B receptor (hNMB-R) and the gastrin-releasing peptide receptor (hGRP-R) [54,55]. These three GPCRs have since been noted to bind bombesin-like peptides. Interestingly, so far, no endogenous high-affinity ligand has been identified for hBRS-3. However, several agonist and antagonist molecules have been synthesized and characterized [55,56]. hBRS-3 is thought to play several roles in lung development, tumor growth, and key endocrine functions such as energy metabolism, glucose homeostasis, and food intake [55].

Lammercih et al. reported the purification and identification of two forms of hemorphin-7, VV-hemorphin-7 and LVV-hemorphin-7, from the human placenta that triggered agonistic action on hBRS-3 [53]. Indeed, both VV-hemorphin-7 and LVV-hemorphin-7 induced a dose-dependent activation of hBRS-3 when overexpressed in CHO cells as well as in a small cell lung cancer cell line (NCI-N417) endogenously expressing the receptor [53]. The biological activity of VV-hemorphin-7 and LVV-hemorphin-7 on hBRS-3 was demonstrated in CHO-hBRS-3 expressing cells as well as NCI-N417 cells with intracellular Ca^2+^ release via the activation of the heterotrimeric G_α16_ protein [53]. The study showed a dose-dependent hemorphin-induced Ca^2+^ release with EC_50_ value around 45 μM for VV-hemorphin-7 and 183 μM for LVV-hemorphin-7 in CHO cells and 19 μM for VV-hemorphin-7 and 38 μM for LVV-hemorphin-7 in NCI-N417 cells [53]. Structure-function analysis reported that the N-terminal valine as well as the C-terminal phenylalanine residues of these hemorphin peptides were important for their activity [53]. In fact, the V-hemorphin-7 peptide (with only one N-terminal valine) showed very weak Ca^2+^ response even at higher concentration (100 μM) while VV-hemorphin-6 (without the C-terminal phenylalanine) completely lost its biological activity via the hBRS-3 in both CHO and NCI-N417 cells [53]. Moreover, the hemorphin-dependent Ca^2+^ response via hBRS-3 was inhibited by either a phospholipase C (PLC) inhibitor (U73122) or an IP_3_-receptor inhibitor (xestospongin C), indicating the activation of the canonical hBRS-3/G_α16_ protein/PLC/IP_3_ pathway [53]. VV-hemorphin-7 also triggered the activation and phosphorylation of ERK1/2 but this was not associated with any significant proliferative effects [53]. Finally, the combined treatment of cells with EC_50_ doses of the synthetic high-affinity ligand [DPhe6,βAla11,Phe13,Nle14]-bombesin(6-14)(or BP9) of hBRS-3 and VV-hemorphin-7 did not result in any potentiation of the biological response ruling out any possible allosteric interaction between these two ligands on hBRS-3 [53]. However, the mechanism of hemorphin-mediated hBRS-3 activation and signaling in physiology is still unknown and is, therefore, a topic worth exploring.

## 6. Hemorphins and the Oxytocin Receptor

Several in vivo studies suggested an indirect link between the effects of hemorphins and oxytocin and its receptors [57,58,59]. Firstly, angiotensin IV (AngIV), via its receptor know as insulin-regulated membrane aminopetidase (IRAP; also known named as AT4R), has been shown to increase oxytocin levels in the rat amygdala [58]. This was associated with remarkable anxiolytic-like effects in rat and mouse models [58]. Coincidentally, the activation of AT4R by LVV-hemorphin-7 triggered a similar anxiolytic-like effect and behavioral responses as oxytocin [58]. Along these lines, AngIV, LVV-hemorphin-7, and oxytocin have all been shown to cause significant antiallodynia in mice, further supporting the link between these peptides [57]. Another recent study by da Cruz et al. reported in vivo evidence for LVV-hemorphin-7 promoting antidepressant and anxiolytic effects that were partly mediated by the oxytocin receptor [59]. Indeed, such LVV-hemorphin-7-mediated behavioral effects were decreased by the oxytocin receptor antagonist, atosiban [59]. Interestingly, the shorter form LVV-hemorphin-6 also promoted similar behavioral effects independently on the oxytocin receptors [60].

Thus, these in vivo studies support the link between hemorphin peptides and oxytocin in the spinal, neuroendocrine, and cardiovascular systems. Overall, data establishes the role of LVV-hemorphin-7/AT4R/oxytocin receptor axis in different behavioral effects observed in rat and mice models. However, there is no evidence of direct binding and activation of oxytocin receptors by hemorphin peptides. The plausible implication of hemorphins in other oxytocin-dependent physiological and pathophysiological responses such as reproduction, labor, breastfeeding, and social and affective behaviors, is also not known. This constitutes an interesting aspect to be investigated in the future.

## 7. Hemorphins and the MAS-Related G Protein-Coupled Receptor X1

The MAS-related G protein-coupled receptor X1 (MRGPRX1) was initially considered as an orphan primate specific receptor expressed in the sensory system and involved in pain and itch [61,62,63]. MRGPRX1 responds to diverse ligands including peptide fragments from precursor proteins such as angiotensin and proenkephalin [64,65]. In addition, MRGPRX1 has been shown to be activated by hemorphins in vitro [66]. Indeed, Karhu et al. isolated two forms of hemorphins, LVV-hemorphin-7 and VV-hemorphin-7, from human platelets that pharmacologically activated MRGPRX1 stably expressed in HEK293 cells by promoting a dose dependent intracellular Ca^2+^ mobilization [66]. The EC_50_ values of LVV-hemorphin-7 and VV-hemorphin-7 were around 8 and 4 μM, respectively, which is consistent with the potency of different hemorphin peptides on other GPCRs described here [66]. In contrast, the synthetic LVV-hemorphin-5 did not trigger any Ca^2+^ response via MRGPRX1 [66]. This further supports the conclusion that, in this context, the C-terminal phenylalanine residue of hemorphins is critical for their biological activity as shown for other GPCRs such as hBRS-3 [53].

The targeting of MRGPRX1 by LVV-hemorphin-7 and VV-hemorphin-7 in vitro using HEK293 cells suggests a role for these peptides in pain perception. However, further investigations in vivo are needed to conclusively establish this. Interestingly, like with MRGPRX1, hemorphins also bind to opioid receptors to modulate analgesia [6,7,8,9,10,11,12]. On the other hand, the MRGPRX1 ligand BAM (1–22) was shown to bind to opioid receptors and this binding was blocked by opioid receptor antagonist naloxone [65]. This suggests a link and/or crosstalk between MRGPRX1 and opioid receptors in producing the analgesic effects of hemorphins.

## 8. Multiple Modes of Action of Hemorphins on GPCRs

It is now evident that hemorphins target a diverse range of GPCRs with implication in multiple physiological and pathophysiological situations (Table 1). The common feature of these GPCRs is that they are all receptors for peptide hormones belonging to rhodopsin-like sub-family. In addition to the existence of various forms of hemorphins, their multiple modes of action on the GPCRs constitute another interesting aspect in their pharmacology (Table 1). Indeed, hemorphins were mainly reported to act as agonists on their GPCR targets including the opioid receptor, the bombesin receptor (hBRS-3), and the MRGPRX1. This was demonstrated either by binding experiments showing competitive binding of hemorphins at the orthosteric binding site of their endogenous ligands [6,26,39,40,41], or by functional assays reporting the direct activation of the downstream receptor-mediated signaling by hemorphins such as the activation of the heterotrimeric G protein, GTPγS incorporation, second messenger production, ERK1/2 phosphorylation, and calcium release [29,39,53,66]. Overall, the affinity and potency of the different hemorphin peptides for their respective receptor targets were within the μM range, which are consistent with the circulating hemorphin levels measured in vivo [14,15,16,17]. However, some studies have not demonstrated the direct binding of hemorphin peptides on the receptors studied. Also, little is known about the structure-function features of GPCR-hemorphin pairs. For instance, it would be interesting to compare the hemorphins with some known opioid peptides such a, DAMGO and some analogs (DALDA, KGOP01) that have recently been studied [67]. This might represent a blueprint to modify hemorphins to change their pharmacological profile in terms of potency/efficacy and selectivity toward the different opioid receptor subtypes. Interestingly, our recent studies revealed, for the first time, a positive allosteric modulation of AT1R by LVV-hemorphn-7 as shown in vitro and in silico [44]. This may be true for other GPCRs such as the bradykinin receptor since hemorphin peptides have been shown to potentiate the bradykinin-mediated hypotensive response in vivo [15]. The dual positive action of hemorphins on the receptors for AngII and bradykinin is very interesting due to the opposite role of these two key hormones of RAS and KKS, respectively, in the control of blood pressure. Moreover, we observed a partial agonistic action of LVV-hemorphin-7 on AT1R-mediated ERK1/2, but not Gαq/IP1 activation, in HEK293 cells, suggesting the implication of a biased effect of this hemorphin on AT1R. So far, there is no evidence for any negative pharmacological effect (antagonistic or negative allosteric modulation) of hemorphins on GPCRs but this may exist for other hemorphin-GPCR pairs that would be identified in the future.

## 9. Conclusions

Here, we provide an overview of the targeting of one of the most important cell surface receptor families, GPCRs, by hemoglobin-derived peptides named hemorphins (Table 1). Initial studies on the biological activity of hemorphins as well as their pharmacological effects on GPCRs (opioid receptors) date back to the end of nineteen eighties. However, the focus later shifted to the effects of these peptides on key enzymes and proteins in several systems (neuroendocrine, sensory, vascular and renal systems). Only a handful of studies have investigated the role of these peptides in the context of GPCRs. Here, we have attempted to revisit this, emphasizing on the targeting of GPCRs by hemorphins as potential components in both physiology and pathophysiology. Diverse roles and modes of action have been established both in vitro and in vivo, but for some hemorphin-GPCR pairs further investigation and validation in more integrated systems are required. Many questions remain, especially those related to pleiotropic targets (GPCRs and others) and effects of hemorphins on GPCRs, which raises the problem of specificity and selectivity. Further structure-function studies would assist in addressing some of these questions. Recent GPCRs identified as targets for hemorphins should open a new era for future studies on other key GPCRs known for their role in major systems (vascular, renal, and neuroendocrine systems). Additionally, the contribution of hemorphins and GPCRs in pathophysiology constitutes an important aspect to be studied in the future. Indeed, it would be interesting to establish a rationale link between the concentrations of the hemorphin peptides in the different pathological situations and the activity/function of the targeted GPCRs. Finally, the diversity of hemorphins along with the multiplicity and complexity of GPCR signaling and pharmacology should open an exciting era in the investigation of these ligand-receptor pairs in the “hot” context of biased signaling/functional selectivity of GPCRs.

**Table 1:** Summary of the different GPCRs targeted by hemorphins.

GPCR	Pharmacology	Model	Binding	Signaling	Physiological Implication	References
Opioid receptors (μ-, κ-, and δ-)	Agonist Antagonist?	in vitro, in vivo and in silico	Competitive radioligand binding assay in rat brain membranes Molecular docking and molecular dynamics	[^35^S]-GTPγS assay (Gi/o coupling?)	Analgesic effects	[6,7,8,9,22,23,26,37,38,39,40,41]
Angiotensin II receptor (AT1R)	Positive allosteric modulator Partial agonist?	in vitro using HEK293 cells and in silico	Nano-BRET binding assay in HEK293 cells Molecular docking and molecular dynamics	BRET with Gαq protein and β-arrestin 2. Gαq/IP1 ERK1/2	Implication in RAS and in the control of blood pressure?	[29,44]
Bombesin receptor (hBRS-3)	Agonist	in vitro using CHO and NCI-N417 cells	Not studied	Gα16/PLC-mediated Ca^2+^ release ERK1/2	Role in the neuroendocrine functions (energy metabolism, glucose homeostasis, and food intake)?	[53]
Oxytocin Receptor	Agonist?	in vivo using rat and mouse	Not studied	Not studied	Antidepressant and anxiolytic effects	[57,58,59,60]
Bradykinin Receptor	Positive allosteric modulator?	in vivo using anaesthetized rats	Not studied	Not studied	Hypotensive effects	[15]
MAS-Related G Protein-Coupled Receptor X1	Agonist	in vitro using HEK293 cells	Not studied	PLC-mediated Ca^2+^ release Crosstalk with the opioid receptors?	Role in pain perception?	[66]

## Data Availability

Not applicable.
